# A meta-analysis of surgical decompression in the treatment of diabetic peripheral neuropathy: Retraction

**DOI:** 10.1097/MD.0000000000012964

**Published:** 2018-10-19

The article, “A meta-analysis of surgical decompression in the treatment of diabetic peripheral neuropathy”,^[1]^ which appeared in Volume 97, Issue 37 of *Medicine*, is being retracted for plagiarism.
